# A High Estimated Prevalence of Onychomycosis Exists Among Danish Children

**DOI:** 10.1111/myc.70129

**Published:** 2025-11-10

**Authors:** Tanja Roehmer Wriedt, Lise Heilmann Jensen, Abdullah Mansouri, Kristoffer Nagy Skaastrup, Gregor Borut Ernst Jemec, Maiken Cavling Arendrup, Ditte Marie Lindhardt Saunte

**Affiliations:** ^1^ Department of Dermatology Zealand University Hospital Roskilde Denmark; ^2^ Department of Pediatrics Zealand University Hospital Roskilde Denmark; ^3^ Department of Clinical Medicine, Faculty of Health Sciences University of Copenhagen Copenhagen Denmark; ^4^ Mycology Unit, Department for Microbiology and Infection Control Statens Serum Institut Copenhagen Denmark; ^5^ Department of Dermatology and Allergy Copenhagen University Hospital ‐ Herlev and Gentofte Copenhagen Denmark

**Keywords:** child, onychomycosis, paediatrics, prevalence, tinea

## Abstract

**Background:**

The prevalence of onychomycosis among children is suspected to be increasing. The current global prevalence of paediatric onychomycosis ranges from 0% to 7.7%. Clinical observations in Denmark suggest the same but to our knowledge no study exists estimating the prevalence of onychomycosis among Danish children.

**Objective:**

The aim of the study was therefore to estimate this prevalence.

**Methods:**

Children and their siblings were included upon visiting the Paediatric Department, Zealand University Hospital, Roskilde, Denmark. The children and their legal guardian, if under the age of 15 years, were asked to answer a questionnaire, and the children had their finger‐ and toenails photographed. Children with nail abnormalities suggestive of onychomycosis were offered a referral to the Department of Dermatology, Zealand University Hospital, Roskilde, Denmark for clinical and mycological examination.

**Results:**

A total of 170 children with a mean age of 6.9 years were included. Ninety‐seven (57.1%) were boys and a total of 46.5% of the children were healthy. Twenty‐nine children had nail abnormalities, and 23 accepted a referral to the Department of Dermatology. Four children had onychomycosis, all caused by *T. rubrum* , resulting in an estimated prevalence of 2.4% (CI 0.6%–5.9%).

**Conclusion:**

The estimated prevalence (2.4%) of onychomycosis among Danish children is higher than expected compared to other European countries, but larger studies are needed to validate these findings. This supports the suggestion of an increasing prevalence of paediatric onychomycosis.

## 1 Introduction

Onychomycosis, a fungal nail infection, is the most prevalent nail disease, with an estimated global prevalence of 10% in the adult population [1]. The prevalence among children varies depending on geographical region with a global prevalence ranging from 0% to 7.7% [2, 3]. It is estimated that 15% of nail dystrophies in children are caused by onychomycosis in contrast to 50% in adults [4, 5]. Several studies from multiple countries suggest that onychomycosis among children is increasing [2, 4, 6, 7, 8, 9]. The lower prevalence of onychomycosis in children, as compared with adults, can be explained by several factors. Children are known to have a good peripheral blood supply, a history of fewer nail traumas, faster outgrowth of the nail plate, and they have probably been exposed less to environments with fungal elements [4]. General risk factors for developing tinea unguium are increasing age, male sex, warm and humid environment, using public bathing facilities and comorbidities such as diabetes, vascular disorders, Down syndrome and immunosuppression [10].

Childhood onychomycosis mostly affects toenails distally and laterally [2, 4, 11, 12]. It usually progresses from tinea pedis to the nail plate [13, 14], but the infected nail can also act as a reservoir for tinea pedis [15]. The most common pathogens causing onychomycosis are dermatophytes, especially *Trichophyton* (*T*.) *rubrum* [2, 16]. A study found that non‐dermatophyte onychomycosis in children younger than 3 years is primarily caused by *Candida* (*C*.) *albicans* [8], but *C. glabrata* has also been described in the literature [2]. The authors do not specify if it is finger or toenails, but a correlation between finger sucking and onychomycosis of the fingernails caused by Candida species has been shown [13].

Early diagnosis and treatment of onychomycosis are key to minimising societal spread. Clinical observations suggest an increased prevalence of onychomycosis among Danish children. However, to our knowledge, no systematic studies of the prevalence of onychomycosis among children exist.

## 2 Method

This study is cross‐sectional and includes hospitalised children and their siblings visiting the Paediatric Department, Zealand University Hospital, Roskilde, Denmark. Inclusion criteria were age between 4 weeks and 18 years, resident in Region Zealand, Denmark and a signed informed consent by the legal guardian or the patient (if the patient was ≥ 15 years). Children were excluded if they were referred for onychomycosis.

A questionnaire regarding the child's background information (name, sex, age, and their central person register number (CPR)), familial predisposition to skin diseases (psoriasis, atopic dermatitis, fungal infections), former dermatological diseases (e.g., atopic dermatitis, psoriasis, tinea), and current health status including immunosuppression was completed by the legal guardian and/or the child (if ≥ 15 years old) with assistance from a healthcare professional. All study participants had their finger‐ and toenails photographed, any nail abnormality was noted, and all photos were evaluated by two independent healthcare professionals (TRW and KNS and in cases of disagreement DMS).

All children with nail abnormalities suggesting onychomycosis were offered a referral to the Department of Dermatology, Zealand University Hospital, Roskilde, Denmark for clinical and mycological examination and treatment. Mycological identification was performed as per routine at the national reference laboratory at Statens Serum Institute, Copenhagen, Denmark [14], using microscopy and culture and polymerase chain reaction (PCR) analysis [17, 18, 19].

Children were treated according to Danish and international guidelines, with terbinafine as the first‐line systemic therapy [20, 21, 22]. Systemic antifungals were given for extensive infection and topical therapy for mild disease. All patients were monitored with routine blood tests and mycological follow‐up (culture) as part of standard dermatological care.

### 2.1 Ethics

The study was approved by the Ethics Committee for Scientific Research in Region Zealand, Denmark (study number SJ‐908 and record number REG‐052‐2021).

### 2.2 Statistics

A sample size calculation was performed using Epitools (https://epitools.ausvet.com.au/prevalencess) with the assumption that the true prevalence of onychomycosis among children is 0.05% with a 95% confidence interval (CI). The calculated sample size required was 164 children. RStudio version 2022.12.0 was used to perform the statistical analysis. The prevalence of onychomycosis was calculated using binomial tests.

## 3 Results

A total of 170 children were included from April 2021 to January 2023. The mean age was 6.9 years and 97 (57.1%) were boys. A total of 46.5% of the children included were healthy according to the questionnaire, while the rest were either sick or answered ‘other’ which was often used if the child had no diagnosis and was being assessed by the doctors at the paediatric department. Only one pair of siblings was included, a brother and a sister.

A total of 29 children had nail abnormalities indicating onychomycosis and 23 of them accepted a referral to the Department of Dermatology, Zealand University Hospital, Roskilde, Denmark. Four children were diagnosed with toenail onychomycosis (Table 2). The prevalence of onychomycosis among children was estimated to be 2.4% (95% CI: 0.6%–5.9%). Of the four children with onychomycosis three were male and the average age was 10 years (6–13 years). All were healthy except one who suffered from a rare genetic abnormality affecting the nervous system. One of the four had previously suffered from a dermatological disease (eczema). None reported hereditary dispositions. All nail abnormalities were located on the toenails, especially toenail numbers 3–5 (Table 1).

**TABLE 1:** Children's demography and clinical characteristics.

Characteristics (%)	Children included (*n* ^a^ = 170)
Male sex	97 (57.1%)
Mean age, years (IQR^b^)	6.9 (2–11)
The child's current condition	
Healthy	79 (46.5%)
Sick	69 (40.6%)
Asthma/allergy	7 (4.1%)
Gastrointestinal diseases	3 (1.8%)
Diabetes type one	21 (12.4%)
Infection	17 (10%)
Urogenital disease	3 (1.8%)
Motor apparatus disease	1 (0.6%)
Neurological disease	5 (2.9%)
Genetic abnormality or syndrome	5 (2.9%)
Psychiatric disease	2 (1.2%)
Other endocrine disorder	2 (1.2%)
Autoimmune disease	2 (1.2%)
Unknown/undiagnosed	2 (1.2%)
Dermatologic condition^c^, ^d^	5 (2.9%)
Other	22 (12.9%)
Undiagnosed	15 (8.8%)
Conditions parents did not regard a disease	5 (2.9%)
Previous dermatologic disease	45 (26.5%)
Atopic dermatitis	13 (7.6%)
Psoriasis	4 (2.4%)
Tinea	3 (1.8%)
Fungal infections^e^	4 (2.4%)
Other dermatologic diseases	24 (14.1%)
Hereditary predispositions to dermatologic diseases	70 (77.8%)
Psoriasis	37 (52.9%)
Eczema	41 (58.6%)
Immunosuppression	5 (7.1%)
Innate	3 (4.3%)
Acquired	0
Unknown	2 (2.9%)
Diabetes	27 (38.6%)
Fungal infections in immediate family members	20 (11.8%)
Children with onychomycosis nail abnormalities	29 (17.1%)
Fingernails	1 (0.6%)
Toenails	29 (17.1%)
Both finger& toenails	1 (0.6%)
Children accepting referral for clinical and mycological examination and treatment	23 (13.5%)
Children tested positive for onychomycosis	4 (2.35%)

^a^ *n* = number of children included.

^b^ IQR: Interquartile range.

^c^ Including conditions from both “other” and “sick” children in the questionnaire.

^d^ Hemangioma, Pachyonychia congenita, hives, seborrheic dermatitis and Henoch‐Schönlein purpura.

^e^ Oral *Candida* infection, onychomycosis, *Candida* intertrigo.

### 3.1 Mycology

*T. rubrum* was the pathogen in all four cases (Table 2). One additional child (ID number 161) had fungal hyphae detected microscopically, but culture and dermatophyte PCR were negative. The test was repeated after 2 months where no hyphae were detected, and the sample was again culture and PCR negative. When examined clinically a suspicion of nail psoriasis with colonization of an unspecified fungus was raised. The child was therefore registered as a child without onychomycosis.

**TABLE 2:** Characteristic of children with onychomycosis and mycology results.

Children ID	9	60	115	123
Sex	Female	Male	Male	Male
Age, years (mean 9.75)	10	10	6	13
The child's current condition	Healthy	Healthy	Healthy	Rare genetic neurologic disorder
Previous dermatologic disease	None	Eczema	None	None
Hereditary dispositions	None	None	None	None
Location of nail abnormalities	Toenails L3‐L5	Toenails R4‐R5	Toenail L4	Toenails R4 and L4
Microscopy	Fungal septate hyphae	Fungal septate hyphae	Negative	Fungal septate hyphae
Culture	*T. rubrum*	Negative	Negative	Negative
Dermatophyte PCR	*T. rubrum*	*T. rubrum*	*T. rubrum*	*T. rubrum*

Abbreviations: ID, identification number; L, left; PCR, polymerase chain reaction; R, right; *T*., *Trichophyton*.

## 4 Discussion

The prevalence of onychomycosis was 2.4% (95% CI: 0.6%–5.9%), but may be an underestimate for children in general as the prevalence is increasing with age and the mean age of children in this study was only 6.9 years (inter quartile range at 2–11 years) [11, 12, 23]. Furthermore, children under the age of 2 years rarely have onychomycosis [24]. A systematic review by Vestergaard‐Jensen et al. found that the prevalence of paediatric onychomycosis is increasing and that the latest estimates for paediatric prevalence are much lower (range 0%–0.2%) in other European countries compared to the prevalence found in this study among Danish children [2]. The higher prevalence of toenail onychomycosis as compared with fingernails is in alignment with other studies [2, 12].

When including children from the paediatric department, siblings of these children were also offered a chance to participate in the study. This could affect the prevalence given that the risk of having onychomycosis increases if a family member has the infection [4, 12]. However, only one pair of siblings was included, and they were screened negative. Other potential limitations are that the included children had contact with the healthcare system and therefore, to some extent, had affected health which could influence the prevalence of onychomycosis estimated in this study. The study made use of a screening questionnaire with self‐reported data risking biases, including selective memory and recall bias. Finally, the sample size was relatively small, resulting in a limited number of children with onychomycosis. Consequently, the scope of statistical analysis was restricted, as conducting a *χ* ^2^‐test was not feasible in a statistically responsible manner.

It was unexpected to find that none of the children with type 1 diabetes had onychomycosis, despite it being known to be a risk factor in the general population [10]. This finding raises the possibility that diabetes may not be significant for paediatric onychomycosis. A similar conclusion was reported in another study examining onychomycosis in 59 children, in which no association between fungal infection and diabetes was identified [13].

Comparing the results with the prevalence of onychomycosis among Danish adults, the prevalences are surprisingly similar. Twenty years ago Svejgaard et al. estimated the prevalence of onychomycosis among Danish adults to be 4.9%, where the majority were caused by dermatophytes [25]. Given the high prevalence of onychomycosis among children estimated in this study and that the global prevalence for adults is currently estimated to be 10% [1], one would also expect an increased prevalence among Danish adults as well.

Children diagnosed with onychomycosis were all infected with the dermatophyte *T. rubrum* . Dermatophytes are the most prevalent cause of onychomycosis (and tinea pedis) in both children [2, 12, 16] and adults [26, 27]. In the adult Danish population the prevalence of tinea pedis is estimated to be 4.1% [25]. This indicates that the mycological findings correlate between adults and children.

In conclusion, our study revealed a prevalence of onychomycosis among Danish children at 2.4% (95% CI: 0.6%–5.9%). The prevalence seems high compared to previously published prevalences in other European countries [2], but larger studies are needed to validate these findings.

## Author Contributions

**Tanja Roehmer Wriedt:** writing – review and editing, investigation, writing – original draft, data curation, formal analysis, software. **Lise Heilmann Jensen:** conceptualization, investigation, writing – review and editing, supervision, visualization. **Abdullah Mansouri:** conceptualization, investigation, writing – review and editing, visualization. **Kristoffer Nagy Skaastrup:** conceptualization, investigation, funding acquisition, writing – review and editing. **Gregor Borut Ernst Jemec:** conceptualization, investigation, writing – review and editing, visualization. **Maiken Cavling Arendrup:** conceptualization, investigation, methodology, writing – review and editing, visualization, resources. **Ditte Marie Lindhardt Saunte:** conceptualization, investigation, funding acquisition, writing – review and editing, writing – original draft, supervision, project administration, validation, visualization, data curation.

## Conflicts of Interest

L.H.J., A.M., K.N.S.: declare no conflicts of interest. G.B.E.J.: Honoraria from AbbVie, Moonlake, Novartis, UCB, and Union Therapeutics for participation on advisory boards, and grants from Abbvie, CSL, Inflarx, Janssen‐Cilag, Leo Pharma, Moonlake, Novartis, Regeneron, Sanofi and for participation as an investigator. He has also received unrestricted departmental grants from Novartis. M.C.A. has over the past 5 years received research grants/contract work (paid to the SSI) from Amplyx, Basilea, Cidara, F2G, Gilead, and Scynexis, and speaker honoraria (personal fee) from Astellas, Chiesi, Gilead, MSD, SEGES and F2G. She is the current chairperson of the EUCAST‐AFST. D.M.L.S.: honoraria as a consultant for advisory board meetings by Sanofi, LeoPharma, Novartis, UCB and as a speaker and/or received grants from the following companies: Galderma, Novartis, Sanofi, Jamjoom Pharma and Leo Pharma during the past 3 years.

## Data Availability

The data that support the findings of this study are available from the corresponding author upon reasonable request.
